# Supplementary material for: Splice-Junction-Based Mapping of Alternative Isoforms in the Human Proteome
Source: Cell Rep. Author manuscript; Available in PMC 2020 Jan 15. (PMC6961840; doi:10.1016/j.celrep.2019.11.026)
Supplement: 2 [file NIHMS1546469-supplement-2.zip › DF1/PXD006675/Aorta/Aorta_13_DPH1_KPVVSGAAEQGGR.pdf]

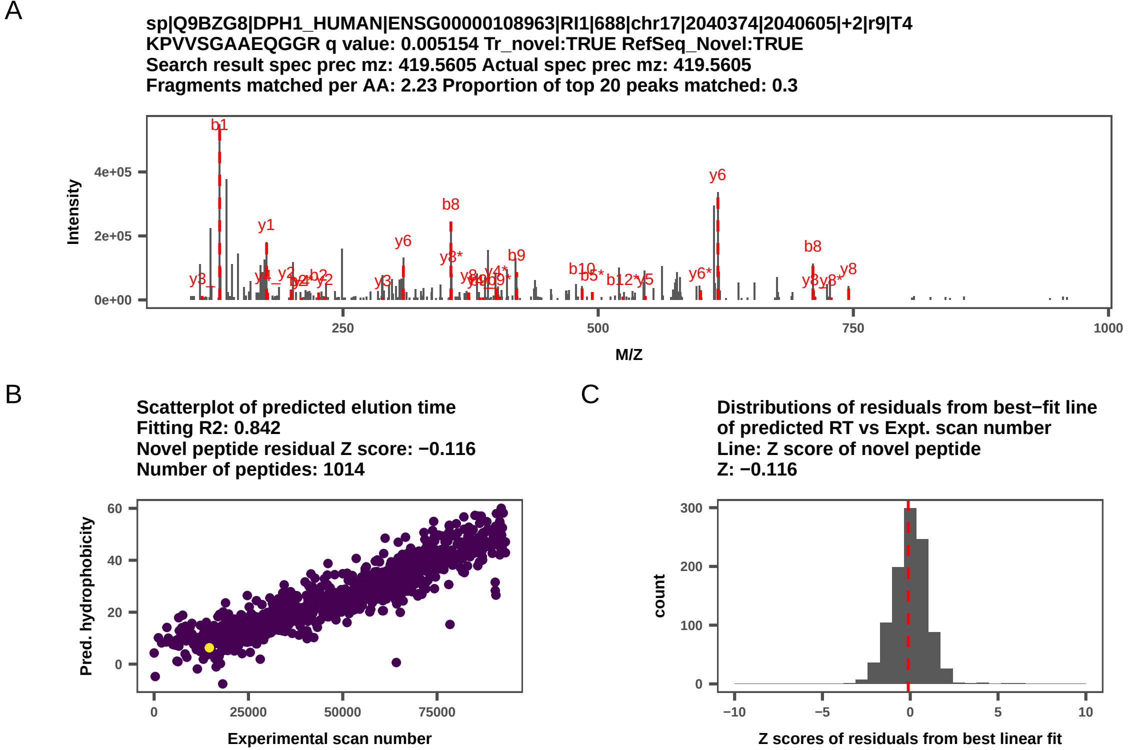
A
sp|Q9BZG8|DPH1_HUMAN|ENSG00000108963|RI1|688|chr17|2040374|2040605|+2|r9|T4
KPVVSGAAEQGGR q value: 0.005154 Tr_novel:TRUE RefSeq_Novel:TRUE
Search result spec prec mz: 419.5605 Actual spec prec mz: 419.5605
Fragments matched per AA: 2.23 Proportion of top 20 peaks matched: 0.3
Intensity
M/Z
B
Scatterplot of predicted elution time
Fitting R2: 0.842
Novel peptide residual Z score: −0.116
Number of peptides: 1014
Pred. hydrophobicity
Experimental scan number
C
Distributions of residuals from best−fit line
of predicted RT vs Expt. scan number
Line: Z score of novel peptide
Z: −0.116
count
Z scores of residuals from best linear fit
